# 

**Published:** 1877-02-20

**Authors:** 


ORIGINAL AND SELECTED ARTICLES—
Cases in Practice. By Geo. A. Dyer, M. D., Washington, Ind. ... 29
Select Formulæ for Diphtheria By G. D. Hodge, M. D., Prescott, Ark ... 30
Bleeding at the Cord. By Dr. A. Petit ... 31
The Therapeutics of Headache. A Lecture Delivered at Bellevue Hospital Medical College. By A. A. Smith, M.D. ... 32
Transfusion and Autotrasfusion Abstract of a lecture delivered by Dr. Lesser before the Berlin Gynæcological Society, December 1, 1874 ... 37

ABSTRACTS AND GLEANINGS—
Treatment of Diphtheria, Differeces Between the Anæsthesia of Ether and Chloroform, 39. Gelseminum as an Antidote to Veratrum Viride, 40. Cool Bathing in the Treatment of Inflammatory Bowel Affections During the Summer, 41. Bromide of Arsenic in the Treatment of Epilepsy. Treatment of Syphylis, 42. Rabies Successfully Treated by Woorara, Treatment of Certain Forms of Acne, 43. Treatment of Typhoid Fever by Ergot. The Cold Douche and Friction in the Treatment of Consumption, 44. Death from Chloroform, in which all the Usual Precautions were taken to Guard Against it, Varicocle—Six Cases Treated by One Method, 45. Propylamine, The Antagonism between Strychnia and Monobromide of Camphor, Inhalation of Iodine, 46. Gastric Ulcer—Milk Treatment, Citrate of Soda in Diabetes, Forcible Dilatation of the Sphincter Ani in the Treatment of Hemorrhoids, 47. A Prophylactic for Sore Nipples, Atropia Poisoning—Dr. Leonard, Smoking Belladonna in Asthma, Emulsion of Phosphorated Oil, 48. A Simple Method of Treating Umbilical Hernia in Infants, Radical Cure for Piles, The Medical Uses of Myrrh, Pilocarpin, 49. The Sudden Checking of Opium Eating, The Use of Conium, Chloral in Ulcers, Prevention of After-Pains, Creosote by Spray, Castor Oil, Vegetable Diet for Epileptics, 50. Valuable Prescriptions, 51. Meteorological Observations, Venous Pulse Resulting from Chloroform, The Microscope, Œnothera Biennis, Value of a Finger, 52. Eczena, Rheumatism Treated with Salicylate of Soda, Important, if True, 53.

EDITORIAL AND MISCELLANEOUS—
Vertebrated Animals in the Human Stomach ... 54
International Regulations—Climate of Upper Georgia—Our Co-Laborers—State Board of Health 55
Exchanging Advertisements ... 56
LITERARY NOTICES ... 56
Deffinitions ... 56

